# A robust treatment planning approach for chest motion in postmastectomy chest wall intensity modulated radiation therapy

**DOI:** 10.1002/acm2.14217

**Published:** 2023-11-29

**Authors:** Yuya Miyasaka, Takuya ono, Hongbo Chai, Hikaru Souda, Sung Hyun Lee, Miyu Ishizawa, Hiroko Akamatsu, Hiraku Sato, Takeo Iwai

**Affiliations:** ^1^ Department of Heavy Particle Medical Science Yamagata University Graduate School of Medical Science Yamagata Japan; ^2^ Department of Radiology Yamagata University Faculty of Medicine Yamagata Japan

**Keywords:** IMRT, PMRT, respiratory motion, robust optimization, setup error

## Abstract

**Purpose:**

Chest wall postmastectomy radiation therapy (PMRT) should consider the effects of chest wall respiratory motion. The purpose of this study is to evaluate the effectiveness of robustness planning intensity modulated radiation therapy (IMRT) for respiratory movement, considering respiratory motion as a setup error.

**Material and methods:**

This study analyzed 20 patients who underwent PMRT (10 left and 10 right chest walls). The following three treatment plans were created for each case and compared. The treatment plans are a planning target volume (PTV) plan (PP) that covers the PTV within the body contour with the prescribed dose, a virtual bolus plan (VP) that sets a virtual bolus in contact with the body surface and prescribing the dose that includes the PTV outside the body contour, and a robust plan (RP) that considers respiratory movement as a setup uncertainty and performs robust optimization. The isocenter was shifted to reproduce the chest wall motion pattern and the doses were recalculated for comparison for each treatment plan.

**Result:**

No significant difference was found between the PP and the RP in terms of the tumor dose in the treatment plan. In contrast, VP had 3.5% higher PTV Dmax and 5.5% lower PTV V95% than RP (*p* < 0.001). The RP demonstrated significantly higher lung V20Gy and Dmean by 1.4% and 0.4 Gy, respectively, than the PP. The RP showed smaller changes in dose distribution affected by chest wall motion and significantly higher tumor dose coverage than the PP and VP.

**Conclusion:**

We revealed that the RP demonstrated comparable tumor doses to the PP in treatment planning and was robust for respiratory motion compared to both the PP and the VP. However, the organ at risk dose in the RP was slightly higher; therefore, its clinical use should be carefully considered.

## 1 PURPOSE

Chest wall postmastectomy radiation therapy (PMRT) is recommended for patients with positive axillary lymph nodes and a high risk of postoperative recurrence. Several studies have reported that PMRT is beneficial in preventing recurrence and improving overall survival.^1^, ^2^, ^3^ A complex treatment plan should be created because PMRT may include the entire chest wall, including the supraclavicular and axillary lymph nodes. Previously, X‐ray irradiation was used with the half‐beam block method, and the target average dose could be improved by combining X‐rays and electron beams.^4^ Intensity‐modulated radiation therapy (IMRT) has been widely used for PMRT in recent years. Several publications have compared IMRT and conventional three‐dimensional conformal radiation therapy (3D‐CRT). For example, Krueger et al. evaluated IMRT, wherein the mean dose in the chest wall is the prescribed dose, and 3D‐CRT, wherein the reference point dose in the chest wall is the prescribed dose.^5^ They showed that the minimum chest wall doses of IMRT and 3D‐CRT were 43.7 ± 11.1 and 31.2 ± 16.5 Gy. Further, IMRT had a significantly improved dose distribution. Rastogi et al. reported significantly lower lung and heart doses with IMRT compared to 3D‐CRT.^6^ Thus, several reports have suggested that IMRT has significant advantages because it improves target coverage and reduces organ at risk (OAR) dose.^5^, ^6^, ^7^, ^8^, ^9^, ^10^

Several studies^11^, ^12^ involving PMRT have demonstrated the need to account for chest wall respiratory movement in the planning workflow. Lizond et al. reported that D_98%_ of PTV decreases by approximately 10% when 5 mm of respiratory motion occurs in chest IMRT.^11^ Thus, respiratory motion during chest irradiation may reduce target coverage, and an effective strategy to mitigate this effect is needed. Wang et al. studied patients with breast cancer and reported motion of 1.03 ± 0.48 mm, 0.95 ± 0.36 mm, and 1.38 ± 0.85 mm in the left‐right (LR), anterior‐posterior (AP), superior‐inferior (SI) directions, respectively.^12^ Lowanichkiattikul et al. examined chest wall motion during deep inhale breath‐hold in patients with PMRT.^13^ Their study reported motions of 1.0, 2.5, and 5.0 mm in the mediolateral, SI, and AP directions. Kinoshita et al. studied 17 patients treated with breast radiotherapy and reported respiratory motion of 1.0 ± 0.6 mm, 2.6 ± 1.4 mm, and 1.3 ± 0.5 mm in the LR, AP, and SI directions, respectively.^14^ Thus, an appropriate approach for respiratory motion is necessary in breast and chest wall irradiation, because the irradiated volume is moving. To compensate for the degraded target dose due to the respiratory motion in breast radiation therapy, skin flash and virtual bolus methods have been suggested in IMRT planning,^11^, ^15^, ^16^ while the irradiation field is extended approximately 2 cm outside the body (flash) in 3D‐CRT.^17^, ^18^ Skin Flash is a tool provided by a specific treatment planning system and is limited to facilities that can use it. The advantage of a virtual bolus plan is its use in any facility. However, the treatment planning process is complicated by the need to calculate the dose distribution for an optimized treatment plan on the computed tomography (CT) image dataset with a virtual bolus and then recalculate the dose distribution on CT image data set without bolus using the same MU factors. Additionally, planning becomes difficult because an adequate target coverage and an acceptable OAR dose in compliance with dose constraints in CT scan with a virtual bolus commonly deteriorates on CT scan without a virtual bolus.

Robust planning, which is an optimization method that considers setup errors and reduces change in dose distribution, has attracted attention in recent years.^19^, ^20^ Several studies have reported on the effectiveness of robust planning.^21^, ^22^, ^23^ Miura et al. performed robust planning for laryngeal cancer cases and reported a better clinical target volume (CTV) coverage with the robust plan with a setup error.^21^ Wada et al. used robust planning for patients with prostate cancer and reported improved CTV coverage at acceptable OAR doses, even with setup errors.^22^ Therefore, robust planning can be considered a beneficial technique for making treatment plans robust against setup errors. We hypothesized that a PMRT treatment plan with IMRT that is robust against chest wall movement, including respiratory movement, can be created by setting the expected chest wall movement as the setup error during robust optimization. In particular, a treatment plan that is robust against chest wall respiratory motion and require fewer steps to input robust parameters can be created by considering respiratory motion as setup errors in the optimization process. However, the effectiveness of robust planning for respiratory motion during chest wall irradiation remains unclear. This study aimed to clarify the effectiveness of robust planning in terms of accounting for the respiratory motion for PMRT chest wall irradiation using IMRT.

## 2 MATERIALS AND METHODS

### 2.1 Patient selection

The current study analyzed 20 patients who underwent PMRT from January 2020 to June 2022, without case exclusion criteria. Of the 20 patients, 10 patients received left‐sided and 10 patients received right‐sided treatment. Out of these 20 patients, one patient was treated for recurrence and another patient received treatment to a lymph node that was not resected. In the remaining cases, based on preoperative condition and lymph node dissection, postoperative irradiation was performed to reduce the risk of recurrence. In principle, axillary lymph node levels 1, 2, 3 and supraclavicular nodes are irradiated in addition to ipsilateral chest wall surgery. In total, five of 20 patients received irradiation, which included the internal mammary lymph node. The institutional review board approved this study.

### 2.2 Treatment planning CT acquisition and contouring

Aquilion LB (Canon Medical Systems, Otawara, Japan) was used for treatment planning CT. The CT slice thickness was 2 mm and the voltage and mAs were 120 kV and 300 mAs, respectively. All the patients were immobilized using a vacuum bag (Blue Bag, Elekta AB, Stockholm, Sweden) with the treatment side arm raised in supine position. CT scan images for treatment planning were acquired using helical scanning under free breathing. Each region of interest was contoured by radiation oncologists using MIM version 7 (MIM Software, Cleveland, Ohio, USA) The ROIs for the CTV, level 1, 2, and 3 axillary lymph nodes, and supraclavicular lymph nodes were drawn with reference to the Radiation Therapy Oncology Group Breast Cancer Atlas.^24^ CTV was defined by radiation oncology according to the goals of the patient's treatment, which were to reduce the risk of recurrence, control the development of positive lymph nodes, and provide treatment against recurrence. A 7‐mm margin was added to the CTV to create the planning target volume (PTV). In addition, to create and evaluate treatment plans for the delivery of prescribed doses to CTVs closer to the body surface, additional target volume PTV_eval_ was defined by retracting the PTV by 1 mm from the body surface.

### 2.3 Treatment planning

Volumetric modulated arc therapy (VMAT) was the preferred treatment technique, and Elekta Synergy (Elekta AB, Stockholm, Sweden) was used as the linac. The X‐ray energy was 6 MV for all the treatment plans. Treatment plans were designed in two arcs with rotation angles of 200°−100° for right chest wall cases and 260°−160° for left chest wall cases. RayStation 10A (RaySearch Laboratories, Stockholm, Sweden) was used as the treatment planning system. The prescribed dose was 50 Gy/25 fr. The collapsed cone algorithm was utilized for dose calculations in all treatment plans. Table 1 lists the dose constraints followed by the treatment plan. All treatment plans were created by a single medical physicist. The optimization parameters were individually adjusted for each treatment plan to account for the different target definitions across different planning approaches and to ensure compliance with prescription dose constraints. The dose constraints followed are shown in Table 1. A non‐robust treatment plan for evaluation was designed to prescribe doses for PTV_eval_ and was standardized to have an average PTV_eval_ dose of 50 Gy (PTV plan [PP]). Additionally, the following two treatment plans were designed for robustness evaluation.

**TABLE 1:** Dose constraints for treatment planning.

Structure	Constraint
PTV_eval_	
	D_max_ < 110%
	V_95%_ > 90%
	V_90%_ > 95%
Bilateral lung	
	V_20Gy_ < 25%
	V_5Gy_ < 55%
	D_mean_ < 15%
Heart	
	V_45Gy_ < 30%
Spinal cord	
	D_max_ < 46 Gy

Abbreviations: D_max_, maximum dose; D_mean_, mean dose; V_x%_, the ratio of the volume irradiated by x% or more of the prescribed dose; PTV, planning target volume; V_xGy_, the ratio of volume irradiated with xGy or more.

#### 2.3.1 Virtual bolus plan (VP)

A virtual bolus of 10 mm thickness with mass density equivalent to water (1.0 g/cm^3^) was created in contact with the body surface. Planning target volume (PTV) was created with 7 mm margin added to the CTV. The virtual bolus at least extends 3 mm away from the PTV to support the prescribed dose coverage. The treatment plan was designed to cover the prescribed dose for the whole PTV, including the bolus area outside the body contour. The dose distribution was then recalculated without virtual bolus while all other settings (such as monitor units, MLC segments) were identical. We confirmed compliance with the dose constraints listed in Table 1 for the CT images without a virtual bolus. The dose normalization was set so that, in principle, the prescribed average PTV_eval_ dose was 50 Gy, with a slight adjustment to comply with dose constraints

#### 2.3.2 Robust plan (RP)

Robust optimization in RayStation uses the minimax optimization method, which performs optimization to minimize penalties in possible worst‐case scenarios.^25^ Additionally, minimax optimization does not assume a worst‐case scenario for each voxel. Rather, it optimizes the penalty for physically possible scenarios, thereby providing a dose distribution based on more realistic assumptions for treatment. The following values were entered for each direction as set up uncertainty in the optimization setup: right: 2.6 mm, left: 2.6 mm, anterior: 6.9 mm, posterior: 6.9 mm, superior: 2.5 mm, and inferior: 2.5 mm. This value was set based on the maximum movement during breast irradiation reported by Kinoshita et al.^14^ The robust planning parameters were modified for individual patients to achieve the uniform dose to the PTV whereas the uncertainty settings were kept as same for all the patients. A RP was created to prescribe doses for PTV_eval_ with the robustness parameter set and comply with the dose constraints shown in Table 1. The treatment plan was designed for the prescribe PTV_eval_ doses_._ Moreover, normalization was set; hence, the average PTV_eval_ dose was 50 Gy.

### 2.4 Evaluation

First, we compared the treatment plans created by the three optimization methods presented in the previous section. The evaluated dose‐volume histogram (DVH) parameters were the following dose constraints: PTV_eval_ D_max_, V_95%_, V_90%_, Lung V_20Gy_, V_5Gy_, D_mean_, Heart D_mean_, V_45Gy_, and Spinal cord D_max_. Then, we evaluated the PTV_eval_ dose for each treatment plan for the respiratory motion condition. The beam isocenter was shifted by a specific value for each of the three treatment plans to assume respiratory motion occurrence, and then the doses were recalculated without changing the beam parameters such as MU and MLC segment shapes. This study used the robust evaluation function on RayStation to evaluate chest wall motion. Table 2 shows the input values for the patient setup uncertainty to reproduce the motion in each direction. This value was set with reference to the reports of Lowanichkiattikul et al.^13^ and Kinoshita et al.^14^ The former evaluated chest wall motion in patients receiving PMRT and reported that the mean mediolateral, SI, and AP values were 1.0, 2.5, and 5.0 mm, respectively, in each direction. Additionally, the maximum amount of movement based on the study of Kinoshita et al. determined the amount of movement evaluated in this study. Isocenter shift patterns 1−4 assume an inhalation condition, patterns 6−9 assume an exhalation condition, and pattern 5 assumes no movement. The value of the isocenter shift was determined by multiplying each input value by $1/\sqrt{3}$. This is one of the default settings in the patient setup uncertainty of the RayStation robust evaluation tool. We assumed that each of the input value shifts is compounded. The D_max_ and V_90%_, of PTV_eval_, heart D_mean_, bilateral lung V_20%_ and spinal cord D_max_ were evaluated in each isocenter shift pattern.

**TABLE 2:** Setting parameters for each isocenter shift pattern entered in patient position uncertainty.

Isocenter shift pattern					
		Anterior—Posterior (mm)	Superior—Inferior (mm)	Left—Right (mm)	
Isocenter shift pattern #		All cases	All cases	Right CW cases	Left CW cases
Inhale motion pattern	1	6.9	2.5	−2.6	2.6
	2	5.0	2.0	−2.0	2.0
	3	3.0	1.0	−1.0	1.0
	4	1.5	0.5	−0.5	0.5
Without motion	5	0.0	0.0	0.0	0.0
Exhale motion pattern	6	−1.5	−0.5	0.5	−0.5
	7	−3.0	−1.0	1.0	−1.0
	8	−5.0	−2.0	2.0	−2.0
	9	−6.9	−2.5	2.6	−2.6

Abbreviation: CW, chest wall.

### 2.5 Statistical analysis

Statistical Package for the Social Sciences version 28 (SPSS, Inc, Chicago, Illinois, USA) was used for our statistical analysis. Significant differences were evaluated using the Friedman and Bonferroni tests because normality could not be confirmed by the Shapiro‐Wilk tests in the comparison between the three treatment planning methods and in respiratory motion evaluation. A *p*‐value of <0.05 was considered statistically significant.

## 3 RESULTS

### 3.1 Treatment planning evaluation

The MUs of PP, VP, and RP were 695.4 ± 110.0, 562.5 ± 62.9, and 723.2 ± 107.4 MU, respectively. The delivery times were 194.2 ± 6.3, 183.7 ± 3.1, and 189.2 ± 7.0 s, respectively. For MU, there was no significant difference between the PP and RP (*p* = 0.069). However, the VP was significantly lower than the PP and RP (*p* < 0.01). The delivery time significantly differed among all three treatment plans (*p* < 0.05). The number of segments in all three treatment plans was similar.

**FIGURE 1:**
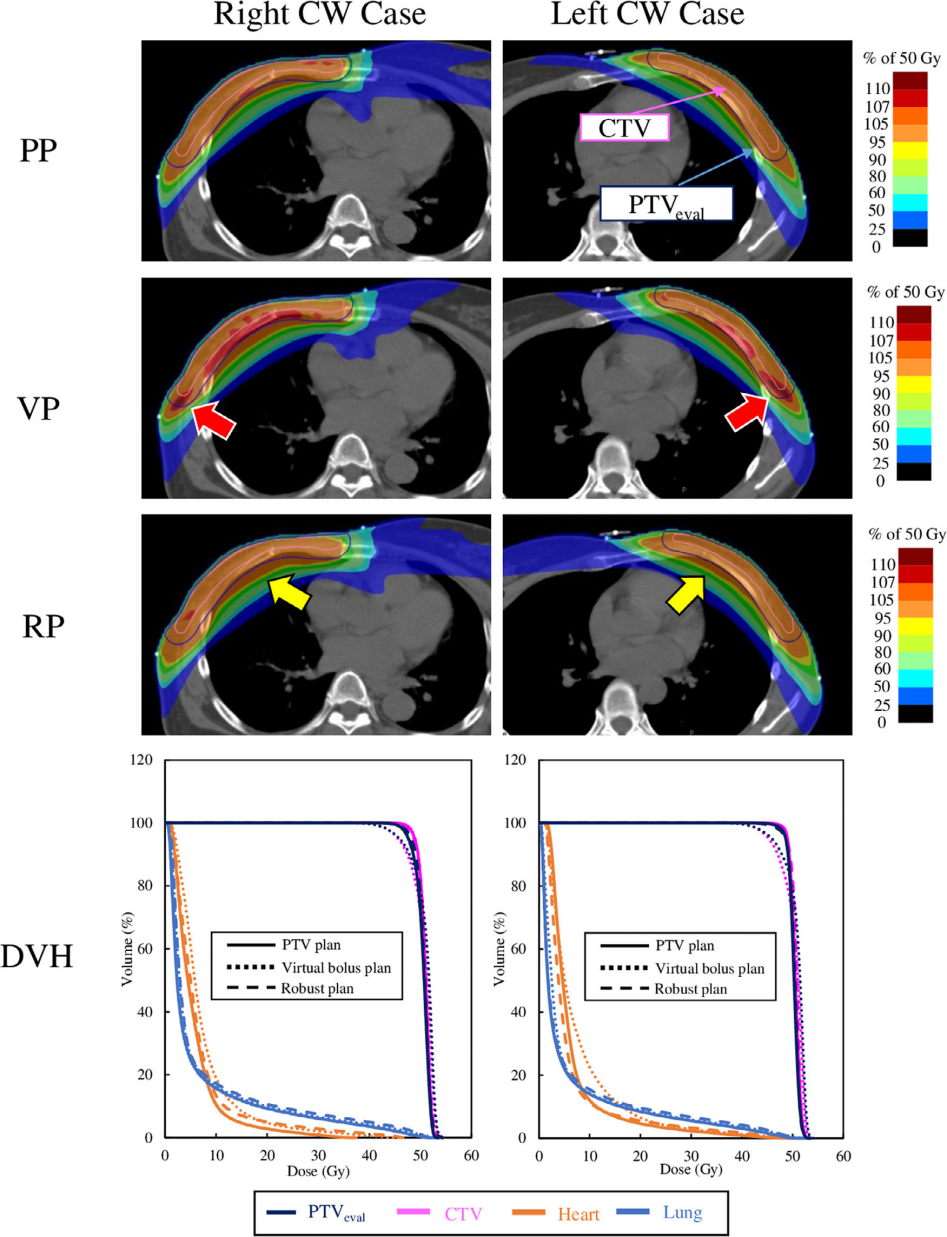
Treatment planning dose distributions and DVH for the three planning methods. In the virtual bolus plan, the red arrows indicate a high dose of more than 107%. In the robust plan, the yellow arrows show the spread of the dose to the lungs. Abbreviations: CTV, clinical target volume; CW, chest wall; DVH, dose volume histogram; PP, PTV plan; PTV, planning target volume; RP, robust plan; VP, virtual bolus plan.

Table 3 shows the comparison of treatment plans of the PP, VP, and RP. There was no significant difference between PP and RP for V_95%_ and V_90%_ (*p* = 0.240 and 0.473, respectively). In contrast, the VP had a significantly lower V_95%_ and V_90%_ than the PP and RP *(p* < 0.01). V_20Gy_ and D_mean_ of the RP were statistically significantly higher than those of the PP for the lung dose. No significant differences were identified among the three treatment planning methods for lung V_5Gy_. V_45Gy_ for the heart dose was significantly higher for the RP than for the other two treatment plans, with a difference of 0.5% from the PP (*p* = 0.022) and 0.3% from the VP (*p* = 0.037). No significant differences were identified in heart D_mean_ among any of the treatment planning methods. The heart D_mean_ of the PP, VP, and RP were 9.4 ± 2.6, 10.0 ± 2.9, and 8.9 ± 2.3 Gy for left chest wall cases, and 5.8 ± 2.1, 6.6 ± 2.2, and 5.8 ± 1.4 Gy for right chest wall cases, respectively. Though the estimated doses for the left chest wall plans were higher in comparison with right chest wall plans, within the treatment side, they were found to be statistically insignificant (*p* > 0.05) among the treatment plans. The mean V_45Gy_ values were 0.2 ± 0.4, 0.6 ± 0.6, and 1.1 ± 0.9 Gy in the PP, VP, and RP, respectively, for left chest wall cases, with significant differences among all treatment plan techniques. For right chest wall cases, the values in all treatment plans were 0.0 ± 0.0 Gy; hence, there were no significant differences. Spinal cord D_max_ was significantly higher in the VP than in the other two treatment planning methods. Figure 1 shows the typical dose distribution and DVH for the treatment plan. The dose distribution of the VP demonstrated a pronounced high dose area. In the PP, the dose was spread to the lung region. DVH comparison revealed a decreased PTV_eval_ and CTV doses with the VP in both left and right cases. No notable differences were observed in lung doses between any of the treatment plans.

**TABLE 3:** DVH parameters of the treatment plan for the three planning methods.

Structure	DVH parameter	PP	VP	RP
PTV	D_max_ (%)	106.8 ± 0.7 (106.5–107.2)	109.3 ± 0.7 (109.0–109.7)	106.8 ± 0.8 (106.4–107.2)
	V_95％_ (%)	95.1 ± 2.4 (94.0–96.3)	90.5 ± 0.5 (90.3–90.8)	96.0 ± 1.1 (95.5–96.6)
	V_90%_ (%)	99.0 ± 0.5 (98.7–99.3)	96.0 ± 0.5 (95.7–96.3)	98.9 ± 0.5 (98.6–99.2)
Lung	V_20Gy_ (%)	8.2 ± 2.3 (7.1–9.4)	9.1 ± 2.8 (7.8–10.4)	9.6 ± 2.6 (8.4–10.8)
	V_5Gy_ (%)	24.4 ± 3.7 (22.6–26.2)	25.0 ± 4.0 (23.1–27.0)	25.6 ± 3.4 (24.0–27.3)
	D_mean_ (Gy)	6.1 ± 1.1 (5.5–6.6)	6.5 ± 1.2 (5.9–7.1)	6.5 ± 1.1 (6.0–7.1)
Heart	V_45Gy_ (%)	0.1 ± 0.3 (0.0–2.4)	0.3 ± 0.5 (0.1–0.6)	0.6 ± 0.9 (0.2–1.0)
	D_mean_ (Gy)	7.6 ± 3.0 (6.2–9.0)	8.3 ± 3.1 (6.8–9.8)	7.3 ± 2.5 (6.2–8.5)
Spinal cord	D_max_ (Gy)	4.8 ± 2.0 (3.9–5.8)	6.1 ± 2.4 (5.0–7.2)	4.7 ± 1.7 (3.9–5.5)

Structure	DVH parameter	*p* value (PP vs. VP)	*p* value (VP vs. RP)	*p* value (PP vs. RP)
PTV	D_max_	<0.001^*^	<0.001^*^	1.000
	V_95％_	<0.001^*^	<0.001^*^	0.240
	V_90%_	<0.001^*^	<0.001^*^	0.473
Lung	V_20Gy_	0.116	0.668	<0.001^*^
	V_5Gy_	1.000	1.000	0.051
	D_mean_	0.185	1.000	<0.001^*^
Heart	V_45Gy_	0.063	0.037^*^	0.022^*^
	D_mean_	0.531	0.045^*^	1.000
Spinal cord	D_max_	0.020^*^	0.019^*^	1.000

*Note*: Each value is the mean ± SD. () indicates 95% confidence interval.

Abbreviations: PTV, planning target volume; D_max_, maximum dose; D_mean_, mean dose; PP, PTV plan; RP, robust plan; VP, virtual bolus plan; V_x%_, the ratio of the volume irradiated by x% or more of the prescribed dose; V_xGy_, the ratio of volume irradiated with xGy or more.

* *p* value < 0.05.

### 3.2 Evaluation of the effects of respiratory motion

**FIGURE 2:**
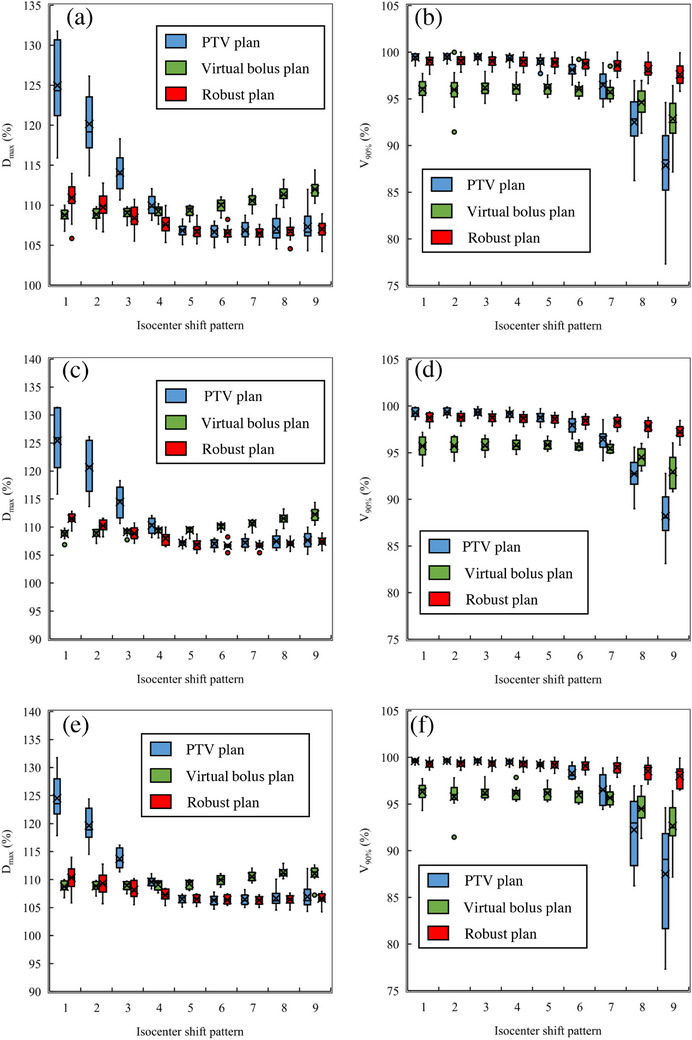
Box‐and‐whisker plots of PTV_eval_ D_max_ and V90% for three planning methods in nine isocenter shift patterns. (a) shows the D_max_ of all cases, (b) shows the V90% of all cases, (c) shows the D_max_ of right chest wall cases, (d) shows the V90% of right chest wall cases, (e) shows the D_max_ of left chest wall cases, (f) shows the V90% of left chest wall cases. Abbreviations: D_max_, maximum dose; PP, PTV plan; PTV, planning target volume; RP, robust plan; V90%, the ratio of the volume irradiated by 90% or more of the prescribed dose; VP, virtual bolus plan.

**FIGURE 3:**
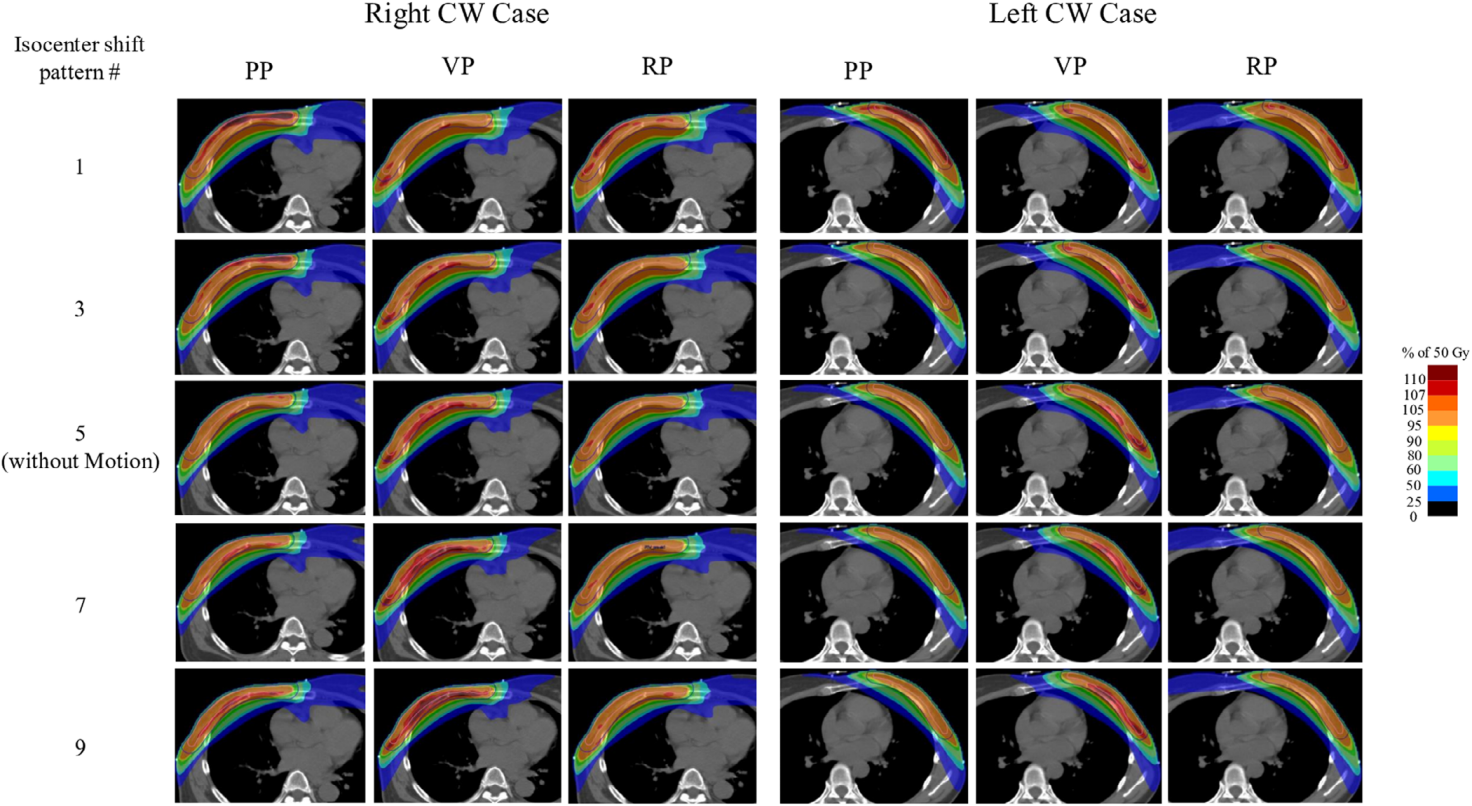
Example of dose distributions for isocenter shift pattern. Abbreviations: CW = cest wall, PTV = planning target volume, PP = PTV plan, VP = Virtual bolue plan, RP = Robust plan.

**FIGURE 4:**
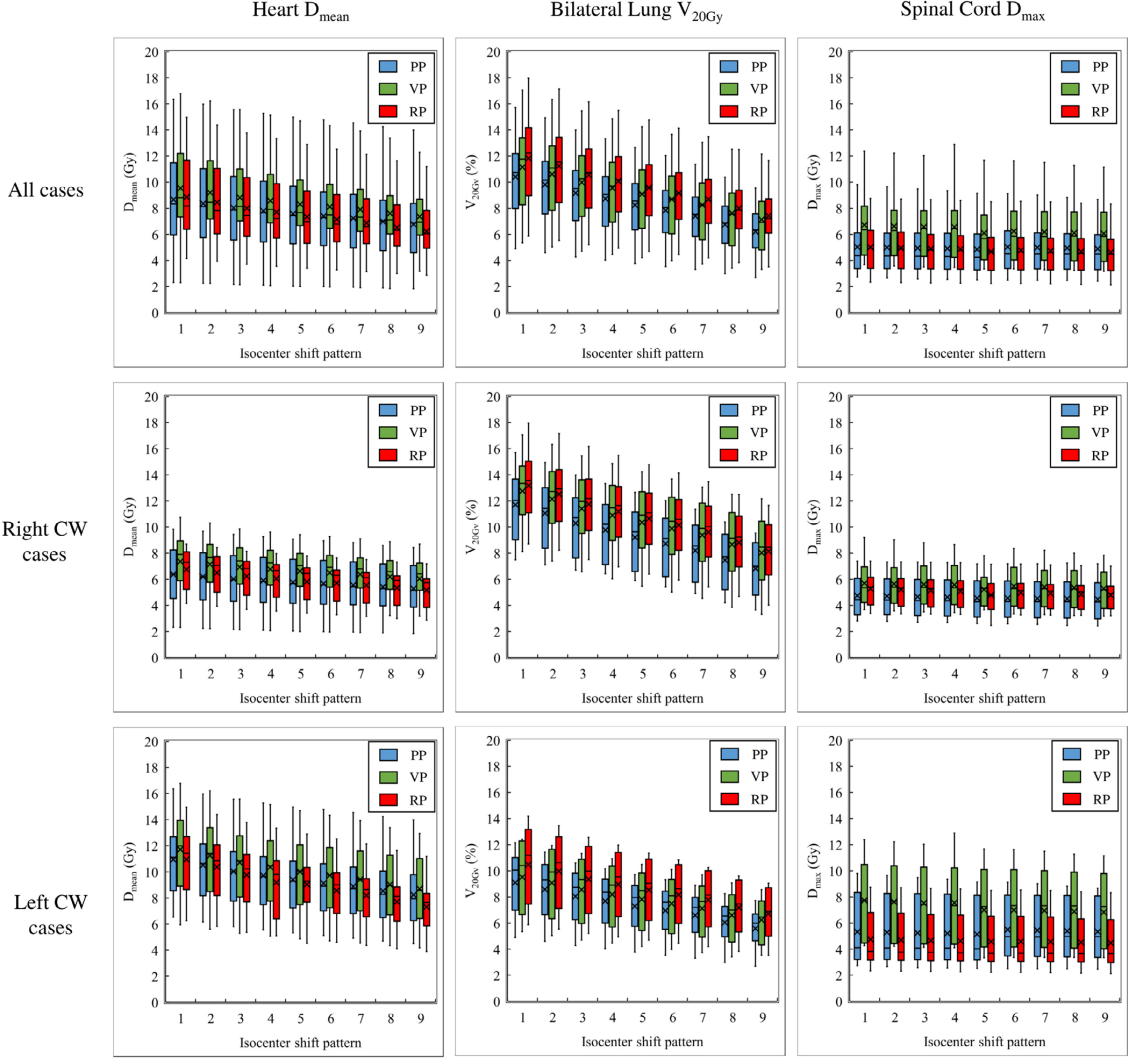
Box‐and‐whisker diagram of Heart Deman, Lung V20%, and spinal cord Dmax for three planning methods in nine isocenter shift patterns. Abbreviations:Dmean, mean dose; Dmax, maximum dose; PTV, planning target volume; PP, PTV plan; VP, virtual bolus plan; RP, Robust plan; V20Gy, the ratio of the volume irradiated by 20 Gy or more of the prescribed dose.

Figure 2 shows the D_max_ and V_90%_ of PTV_eval_ for each isocenter shift pattern. The highest D_max_ in RP was pattern 1 (110.9% ± 1.9%) (Figure 2a). In contrast, the highest D_max_ in the VP was observed in pattern 9, with a mean D_max_ of 112.0% ± 1.2%. The VP demonstrated a significantly higher D_max_ than the RP for exhalation patterns 6−9 (*p* < 0.001). This was similar in both the left and right groups (Figure 2c and e). The D_max_ of the PP was significantly higher for inhalation patterns 1−4 than for the RP (Figure 2a). This trend was similar in both the left and right groups. The highest D_max_ in the PP was pattern 1, with a D_max_ of 125.0% ± 4.9%. The RP for PTV_eval_ coverage demonstrated a smaller reduction in dose to the PP and the VP, especially in patterns 6−9, and a significantly higher V_90%_ compared to the other two treatment planning methods (Figure 2b). The average V_90%_ in the RP for all cases was 98.9% ± 0.5% in the treatment plan, whereas 97.6% ± 1.0% in pattern 9, which showed the lowest V_90%_, which is a change of approximately 1.3%. In contrast, the V_90%_ of the PPs and VPs declined in patterns 6−9. The V_90%_ values of the PP and VP in pattern 9 were 87.9% ± 4.4% and 92.8% ± 2.1%, respectively. This trend was similar for both the left and right groups (Figure 2d and f), with the RP showing a significantly higher V_90%_ than the other two treatment planning methods for patterns 6 through 9 (*p* < 0.05). Figure 3 shows the dose distribution for each pattern for a typical case of the left and right chest walls. No significant high dose areas were observed in any of the isocenter shift patterns in either the left or right chest wall cases. Additionally, PTV_eval_ coverage was not evident. Patterns 1 and 3 showed a pronounced high dose area on the body surface in the PP. The dose coverage of the PTV on the lung side was reduced in patterns 7 and 9. The VP demonstrated a high dose range in patterns 7 and 9. Figure 4 had a box‐and‐whisker diagram of the DVH parameters of the OAR for each isocenter shift pattern. The heart D_mean_ decreased as one went from pattern 1 to 9 in all cases and in left chest wall cases only. The lung V_20%_ was more likely to decrease toward patterns 1−9 in all cases and in both left and right chest wall cases. The spinal cord dose showed little change in isocenter shift pattern from 1 to 9.

## 4 DISCUSSION

We evaluated the robustness of the robust treatment planning methods in PMRT VMAT for respiratory motion and revealed its robustness against the respiratory motion with less variation in both target D_max_ and coverage compared to other treatment planning methods. This revealed that the robust planning method can create robust treatment plans for chest wall respiratory motion in PMRT VMAT.

The RP had similar PTV coverage and maximum dose as the PP in terms of treatment planning. This means that the RP is not inferior to the PP optimized using the conventional optimization method in terms of the target covering. In contrast, the VP had lower target area coverage and a D_max_ than the other two treatment planning methods. Tang et al. created a 1 cm virtual bolus outside the body surface when optimizing the treatment plan as in this study.^15^ They reported that the D_98%_ of the target were 49.662 ± 0.093 and 48.055 ± 0.520 Gy, respectively, compared with the skin flash plan and VP, with a significantly lower target coverage for the VP. This is because the optimization was performed using CT with a virtual bolus, and the final dose calculation and evaluation were performed using CT without a virtual bolus. In our study, a dose reduction was observed, especially on the skin surface, in terms of reduced target coverage (Figure 1). Further, this is due to the shallow depth from the beam injection area and the low dose area of X‐ray buildup overlapping the target area. Lizondo et al. optimized virtual bolus CT values.^11^ They reported that replacing the CT value of the bolus with approximately −500 HU would reduce the difference between the dose distribution at optimization and evaluation. Further, the thickness of the virtual bolus affects respiratory motion treatment plan robustness. The VP could be improved by adjusting the CT value and virtual bolus thickness.

The OAR close to the target, especially the lung and heart doses, tended to be higher in the RP than those in the other two treatment plans. Figure 1 shows the spread of the RP dose distribution toward the lungs compared to the other two treatment plans. This may be because the parameter settings in robust planning. PTV coverage on the lung side was reduced in patterns 7 and 9 in the PP (i.e., conditions in which the chest wall was displaced toward the lungs to simulate expiration). In contrast, the PTV in the RP was covered at the 95% isodose line, even under patterns 7 and 9. This may be caused by extending the dose distribution to the lungs in the RP, as observed in the treatment plan (Figure 1). The dose distribution in the RP was extended to the lung by considering the respiratory motion, resulting in higher lung doses. However, the V_20Gy_ and D_mean_ of the lungs in RP increased by 1.4% and 0.4 Gy, respectively, compared with those in PP. That is, a statistically significant difference was noted. However, these changes in DVH parameters were considered to be slight and clinically acceptable. Additionally, all cases complied with the dose constraints in the RP. Rastogi et al. analyzed the treatment plan for chest wall IMRT and reported a lung V_20Gy_ of 22.09 ± 3.89% and D_mean_ of 11.39 ± 2.4 Gy.^6^ Thus, the lung doses in the RP are not significantly higher than those in conventional IMRT treatment plans. However, careful consideration is needed and the lung dose should be considered in the optimization process in cases with serious pulmonary comorbidities such as pneumonia.

No significant differences were identified between the three treatment plans in the evaluation of cases on each of the left and right chest walls for the heart mean dose. Luo et al. evaluated robust planning for PMRT left chest wall irradiation.^26^ Similar to this study, they reported no significant difference in terms of heart D_mean_ between non‐robust and robust planning. Therefore, the use of RP for irradiation of either the left or right chest wall does not increase the mean dose to the heart. In contrast, the heart V_45Gy_ had significantly higher values in RP. Although not results of the heart dose as we have evaluated, Luo et al. reported that the ipsilateral lung high‐dose parameter, an OAR that is close to the chest wall as the heart, was significantly higher with robust planning. However, it remains unclear if these can evaluate the same high‐dose spread to the lungs and heart side in RP. As shown in the dose distribution in Figure 1, the relatively high dose at approximately 90% of the prescribed dose in RP is spread to the lungs and heart side. This can be attributed to the fact that the chest wall has a wider dose distribution to ensure the target volume dose even in the expiratory direction (i.e., in the dorsal right direction). However, since the heart V_45Gy_ was not elevated in the right chest wall cases, it is not necessary to consider this when irradiating the right chest wall. Since the average increase in the heart V_45Gy_ in RP is approximately 0.5%, it does not have a significant effect. However, the high‐dose spread to the heart should be considered in left chest wall cases.

He et al. evaluated the robustness of treatment planning for the different thicknesses of the virtual bolus in VP.^27^ They reported a reduction of up to 1.05% in D98 for a 5 mm virtual bolus and 1.99% for a 1‐cm virtual bolus when there was a 3‐mm isocenter shift. Based on these results, a 5‐mm‐thick virtual bolus is more robust against chest wall motion than a 1‐cm‐thick virtual bolus for treatment planning. In this study, the VP only considered a 1 cm thick virtual bolus. In our study, the thickness of the virtual bolus was set at 1 cm because a treatment plan with a PTV margin of 7 mm should be established according to our protocol. Optimizing the thickness of the virtual bolus and modifying the PTV margin may improve the robustness of the VP to the chest wall motion beyond what was shown in this study. Nevertheless, this finding should be further investigated.

The RP was the most robust of the three treatment plans, with the least variability in target covering, in terms of chest wall motion robustness (Figure 2b, d, and f). Of all isocenter shift patterns, V_90%_ was > 98% for patterns 1−8, and pattern 9, which had the lowest V_90%_, was 97.6% ± 1.0% in the RP. The RP demonstrated a significantly higher V_90%_ in patterns 6−9 than the PPs and VPs (*p* < 0.05). The V_90%_ of the PPs and VPs had variations of 3.2% and 11.1%, respectively, relative to the plan in pattern 9. This indicates that robust planning was the most robust treatment plan among those examined in this study.

The comparison between the virtual and RP revealed that PTV_eval_ coverage was comparable in patterns 1−4, which are the conditions that reproduced inhalation. In contrast, the RP had significantly higher V_90%_ values of PTV_eval_ than that of the VP in patterns 6−9, which are conditions that simulate exhalation. This indicates that the virtual bolus and RPs are equally robust for inhalation motion; however, the RP is more robust than the VP for exhalation motion. This may be because the VP is optimized with the virtual bolus formed from the body surface to the outside, and does not consider the chest wall moving toward the lungs. The planning CT image used in our study is not a four‐dimensional CT (4DCT) and it represents an average of the expiratory and inspiratory breathing cycle. Therefore, the respiratory motion must be considered for both exhalation and inhalation if respiratory gating CT, such as 4DCT, is not used.

Luo et al. evaluated the effect of treatment planning on the robust optimization of RayStation in PMRT VMAT in the left chest wall.^26^ Results showed that RP was robust to chest wall movement compared with PP, a non‐robust treatment plan, which was similar to the current study. One difference between the Luo et al. and current study, the right chest wall was also evaluated. We have found that robust planning is as robust to chest wall motion in right chest wall cases as it is in left chest wall cases. Moreover, there could be a difference in heart dose between the left and right chest wall cases. Nevertheless, this study found that the mean cardiac dose of the RP did not significantly differ from that of the PP and VP in right chest wall cases.

This is because in a case similar to the chest wall movement in pattern 1 inspiration, the OAR is displaced to the high‐dose side because the chest wall is displaced to expand in the anterior direction. In case of the right chest wall, the heart was far from the high‐dose area, and the chest wall movement could cause minimal changes in the heart. As for the spinal cord, its position was far from either the left or right chest wall, and only a low dose was distributed. Thus, the change in the spinal cord was minimal with respect to the change in the chest wall position. There were no particular differences among the three treatment plans, and the effect on OAR dose for chest wall displacement could occur equally in all treatment plans.

The MUs of the three treatment planning methods were the smallest for VP with a significant difference. Tang et al. showed that the mean MU for the skin flash and virtual bolus treatment plans were 866.0 ± 68.1 and 760.9 ± 50.4 MU, respectively, with a significantly lower MU in virtual bolus treatment plan, which was similar to this study. In the VP, the bolus is a scatterer. Moreover, due to scattered radiation, the dose is higher than that in other treatment plans, which may have resulted in a lower MU compared with that in other treatment plans when normalizing with the same prescription. Further, VP had the shortest delivery time, thereby indicating that virtual bolus treatment plan is the simplest treatment plan with the best throughput. The MU values and delivery time were comparable between PP and RP. Thus, the PP and RP may have a comparable complexity.

In this study, the evaluation of dose changes for the respiratory migration of OARs was only informative. This is because chest wall motion was reproduced by the method of isocenter motion, which does not account for organ deformation. The use of the simulate organ motion tool of RayStation is a method for evaluating OAR doses with a greater accuracy. The simulate organ motion tool was not used in this study because it is a deformable image registration‐based function and its accuracy could not be validated and guaranteed. The application of 4DCT scan is another possible evaluation method. Nevertheless, since 4DCT scan was not obtained in the cases used for validation in this study, evaluation could not be performed. Since IMRT is becoming more mainstream nowadays, it is important to evaluate the effects of respiratory migration via 4DCT scan. Nevertheless, further studies using this approach should be performed to yield more significant results. Although chest wall motion occurs inherently as a random error, it was difficult to analyze in this study and was therefore limited to evaluation as a systematic error. Since it would be ideal to evaluate the impact on random respiratory motion that actually occurs, more detailed studies are desirable.

## 5 CONCLUSION

This study demonstrated the effectiveness of robust planning in PMRT VMAT. Our results proved that robust treatment planning with due consideration to respiratory motion can be achieved using the ‘robust planning algorithm’ from RayStation treatment planning system. The target coverage from RP was similar to the other conventional treatment planning methods. Though there is a slightly higher OAR dose observed from RP, it is clinically acceptable. The application of robust planning should be cautiously considered in cases with complications such as pulmonary conditions.

## AUTHOR CONTRIBUTIONS

Yuya Miyasaka‐writing the manuscript, data analysis; Takya ono‐creating robustness plan and data analysis and manuscript review; Honbo Cahi‐manuscript review; Hikaru Souda‐data analysis and review manuscript; Sung Hyun Lee‐data analysis and review manuscript; Miyu Ishizawa‐review manuscript; Hiroko Akamatsu‐contouring ROIs, clinical integration, clinical review and review manuscript, Hiraku Sato‐clinical integration, clinical review and review manuscript, Takeo Iwai‐management and coordination responsibility for the research activity planning and execution.

## CONFLICT OF INTEREST STATEMENT

The authors declare that they have no conflict of interest.

## Data Availability

Research data are stored in an institutional repository and will be shared upon request to the corresponding author.
